# Doctors' Wills

**Published:** 1914-03-14

**Authors:** 


### Doctors' Wills.

Dr. Joseph Rutter, M.D., of Hove, who died on December 16, aged seventy-nine, left estate of the gross value of £30,665, of which £28,720 is net personalty. The testator left £200 to Dr. Barnardo's Homes.

Mr. George Cronson, of Eastbourne, retired private hotel proprietor, who left estate of the gross value of £17,019, has bequeathed the residue of his estate upon trust, subject to a few other bequests, for his wife for life, and subject to her interest he left £3,600 for charitable purposes, including £1,000 to Dr. Barnardo's Homes and £500 to the Princess Alice Memorial Hospital, Eastbourne.

Mr. Philip Whitcombe, of Gravesend, said to be the oldest doctor in the country, who died on January 24, aged ninety-seven, has left estate of the gross value of £15,321, of which £14,200 is net personalty. Probate of his will has been granted to his sons, Mr. Philip Percival Whitcombe and the Right Rev. Robert Henry Whitcombe, Bishop of Colchester. The testator left his estate to his children in equal shares.

